# Dark brown serum and plasma samples: a case report

**DOI:** 10.11613/BM.2020.021002

**Published:** 2020-04-15

**Authors:** Lora Dukic, Nikolina Maric, Ana-Maria Simundic

**Affiliations:** 1 Department of medical laboratory diagnostics, University Hospital „Sveti Duh“, Zagreb, Croatia; 2 Department of emergency and intensive medicine, University Hospital „Sveti Duh“, Zagreb, Croatia; 3 Faculty of Pharmacy and Biochemistry, University of Zagreb, Zagreb, Croatia

**Keywords:** case report, critical care, extra-analytical phase, sample coloration

## Abstract

This case report describes occurrence of unusual, dark brown coloration of citrate plasma and serum samples in a female 68 years old patient admitted into Emergency department (ED). Patient complained of nausea and vomiting, fever up to 38.9°C, colicky pain in abdomen, diminished urinary output and yellowish skin tone. Her medical history included arterial hypertension, hypothyroidism and facial squamous cell carcinoma. For previous two years, she was treated with tuberculostatic therapy for *Mycobacterium avium* positive interstitial lung disease. Regular follow-up showed no signs of active disease. Upon admission to ED, complete blood count (CBC) analysis showed low red blood count (RBC) (3.76 x10^12^/L (reference interval (RI) 3.86 – 5.08 x10^12^/L)), low haemoglobin (Hb) concentration (111 g/L (RI 119 - 157 g/L)) and low haematocrit (Hct) (0.310 L/L (RI 0.360 – 0.470 L/L)). Biochemistry analytes were high, with foremost lactate dehydrogenase (LD) activity (2900 U/L, RI < 240 U/L). After communication with the clinician, methaemoglobin measured in arterial blood gas sample was reported. Patient was admitted to the Intensive care unit and upon reflex testing of haptoglobin, intravascular haemolysis was confirmed. This case indicates that every case of brown coloration of the serum must be promptly communicated to the clinician. Reflex testing assured timely diagnosis and favourable patient outcome.

## Introduction

Serum or plasma samples from patients admitted to Emergency department (ED) frequently have coloration (*1*). Depending of the underlying cause, red, icteric or milky appearance are most observed discoloration of the serum or plasma after centrifugation of the sample taken for biochemistry or coagulation testing. In most of the cases, red coloration is a result of *in vitro* haemolysis (*2*). Interpretation of biochemistry tests and haptoglobin concentration together with patient medical record can be useful when *in vivo* haemolysis is suspected (*3*). While *in vitro* haemolysis is a consequence of preanalytical errors, icteric and milky serum or plasma samples are pathological (*4*).

There are rare situations when unusual serum coloration is met, *e.g.* green plasma sample coloration due to contrast dye application or more occasionally in women taking oral contraceptives (*5*). Estrogens from contraceptives could increase concentration of the copper-containing ceruloplasmin, thus causing green coloration. Patients having genetic defects related to enzymes involved in formation of bilirubin, could have “green jaundice”, when bilirubin and its intermediate products are found in circulation (*6*). Strawberry pink coloration of blood before centrifuging was described in a 3-month old boy as a consequence of familial combined hyperlipidaemia (*7*).

Here we present a case of a patient with unusual, dark brown coloration of citrate plasma and serum samples. The patient signed an informed consent and institutional Ethical Board approved the publication of this case report. A female 68 years old patient was admitted to the ED, complaining of nausea and vomiting, fever up to 38.9°C, colicky pain in abdomen, diminished urinary output and yellowish skin tone. Her medical history included arterial hypertension, hypothyroidism and facial squamous cell carcinoma. For the previous 2 years, she was treated with the tuberculostatic therapy for *Mycobacterium avium* positive interstitial lung disease. Regular follow up showed no signs of active disease. Eight months after the discontinuation of tuberculostatic therapy, repeated microbiological cultures were negative. She used ibuprofen because of back pain. She did not have a history of renal disease and laboratory tests for renal function were normal. Results of the routine laboratory tests done in the ED showed anaemia, thrombocytopenia and acute renal failure.

## Laboratory analyses

Upon admission, complete laboratory workup was requested: complete blood count, biochemistry tests (aspartate aminotransferase (AST), alanine aminotransferase (ALT), creatine kinase (CK), lactate dehydrogenase (LD), gamma-glutamyltransferase (GGT), alkaline phosphatase (ALP), glucose, urea, creatinine, total and direct bilirubin, serum amylase, sodium, potassium, chloride, C-reactive protein), coagulation (prothrombin time (PT), activated partial thromboplastin time (APTT) and fibrinogen) and arterial blood gases.

Blood samples were taken for complete blood count (ethylenediaminetetraacetic acid (EDTA) vacuum tube), biochemistry testing (vacuum tube with clot activator) and for coagulation testing (sodium citrate vacuum tube). All tubes were from Vacutest Kima (Piove di Sacco, Italy). Arterial sample for blood gas testing was obtained by arterial puncture into the syringe with spray dried balanced lithium heparin (Becton Dickinson and Company, Franklin Lakes, USA).

**Figure 1:**
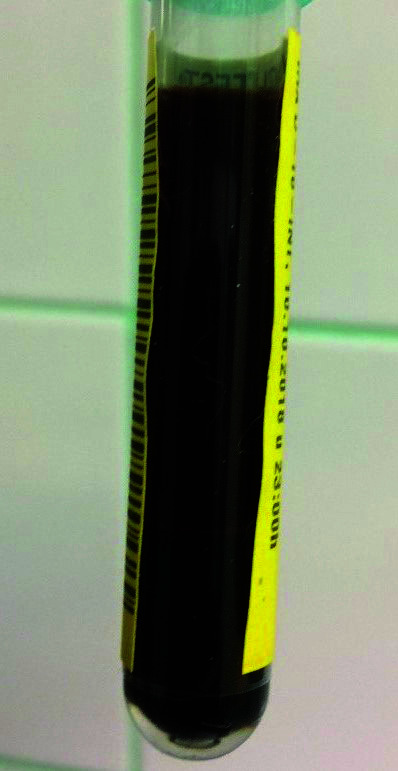
Dark brown citrate plasma sample of a 68 years old female patient

After centrifugation, dark brown coloration of the serum and plasma was noticed (Figure 1). In our laboratory, coagulation tests are performed using the photometric method at 340 nm on BCS XP analyser (Siemens, Marburg, Germany). If plasma is haemolytic, icteric or lipemic, reflex testing is automatically performed on 570 nm, as a routine procedure. In this particular case, plasma sample for coagulation testing was not processed because the coloration of plasma was too intensive, and this was communicated to the clinician. Complete blood count was determined using the Advia 2120i blood cell counter (Siemens Healthcare GmbH, Erlangen, Germany). Biochemistry tests were measured in native and diluted serum sample using system reagents on AU400 analyser (Beckman Coulter Inc, Brea, CA, USA).

## Further investigation

After communication with the clinician, methaemoglobin and haptoglobin were requested, in order to investigate the possible cause of the brown coloration of the patient plasma/serum.

Methaemoglobin and blood gases were measured on blood gas analyser ABL 800 (Radiometer Medical Aps, Bronshoj, Denmark). In our institution serum haptoglobin is done as a routine test and is not available as a stat assay on a 24/7 basis. This is why serum sample taken at admittance was analysed by immunoturbidimetric haptoglobin assay on Architect c8000 biochemistry analyser (Abbott Laboratories, Abbott Park, USA) in our referral laboratory, on the first day of patient hospitalization. Follow-up of haptoglobin concentration was performed in our laboratory, on the second and seventh day of the hospitalization on BN Prospec nephelometer (Siemens, Marburg, Germany).

## What happened

Since methaemoglobin was within the reference range, methaemoglobinemia was excluded as a cause of serum/plasma discoloration. Patient was admitted to the Intensive care unit (ICU) where diagnostic evaluation continued. Anaemia, high activities of LD and indirect hyperbilirubinemia were indicative for *in vivo* haemolysis. Additional laboratory results (represented in Table 1) - reticulocyte count, schistocytes present on the peripheral blood smear done in Cytology department, indirect and direct Coombs tests done in Transfusion department - indirect test result negative, direct test result positive and haptoglobin were done which confirmed *in vivo* haemolysis.

**Table 1:** Laboratory results during hospitalization in a patient with *in vivo* haemolysis

**Analyte**	**Day 1 (ED)**	**Day 2, afternoon (ICU)**	**Day 3**	**Day 7**	**Day 25**	**Reference interval**
**Serum/plasma colour**	Dark brown	Yellow	Light yellow	/	/	Light Yellow
**Haematology tests**						
Leukocytes (x10^9^/L)	21.8	13.5	11.5	9.8	7.1	3.4 - 9.7
Erythrocytes (x10^12^/L)	3.76	3.50	2.98	3.27	2.94	3.86 - 5.08
Haemoglobin (g/L)	111	99	82	94	84	119 - 157
Haematocrit (L/L)	0.310	0.280	0.240	0.270	0.240	0.356 - 0.470
MCV (fL)	82	79	81	83	83	83 - 97
MCH (pg)	29	28	28	29	29	27 - 34
MCHC (g/L)	359	357	341	346	346	320 - 345
RDW (%)	14.1	14.3	14.4	15.2	13.9	9 - 15
MPV (fL)	8.6	8.8	9.4	8.7	8.7	6.8 - 10.4
Platelets (x10^9^/L)	84	72	62	143	181	158 - 424
Reticulocytes (‰)	/	22	/	/	/	5 - 22
**Biochemistry tests**						
AST (U/L)	368	241	49	27	19	8 - 30
ALT (U/L)	73	54	31	29	13	10 - 36
CK (U/L)	238	/	52	45	/	< 153
LD (U/L)	2900	/	490	252	240	< 240
GGT (U/L)	46	/	32	/	/	9 - 35
ALP (U/L)	141	/	61	/	/	64 - 153
Glucose (mmol/L)	6.6	/	/	/	/	4.4 - 6.4
Urea (mmol/L)	15.5	24.6	13.7	20.9	11.0	2.8 - 8.3
Creatinine (µmol/L)	188	370	323	588	94	49 - 90
Total bilirubin (µmol/L)	133.4	/	15.2	11.8	8.2	3 - 20
Direct bilirubin (µmol/L)	29.8	/	3.4	2.6	1.4	< 5.0
Amylase (U/L)	102	/	/	/	/	23 - 91
Sodium (mmol/L)	135	132	139	140	133	137 - 146
Potassium (mmol/L)	4.8	4.3	4.1	4.5	4.5	3.5 - 4.7
Chloride (mmol/L)	100	99	101	102	102	97 - 108
CRP (mg/L)	63.3	/	93.3	59.6	3.6	0 - 5.0
**Blood gas analysis**						
pH	7.394	/	/	7.431	/	7.350 - 7.450
pCO_2_ (kPa)	4.53	/	/	5.86	/	4.70 - 6.40
pO_2_ (kPa)	7.69	/	/	14.40	/	10.00 - 13.40
BE	- 3	/	/	4	/	- 2 to 3
Standard bicarbonate (mmol/L)	21	/	/	28	/	23 - 27
Actual bicarbonate (mmol/L)	20.3	/	/	28.7	/	21.0 - 29.0
Total bicarbonate (mmol/L)	18.7	/	/	27.3	/	23.0 - 27.0
Oxygen saturation	0.89	/	/	0.99	/	0.94 - 0.98
Methaemoglobin (%)	3	/	/	/	/	< 5
**Specific proteins**						
Haptoglobin (g/L) (immunonephelometry)	/	0.32	/	1.48	2.20	0.30 - 2.00
Haptoglobin (g/L) (immunoturbidimetry)	0.15	/	/	/	/	0.63 - 2.73
Immunoglobulin G (g/L)	/	/	8.58	/	/	7.02 - 16.32
Immunoglobulin A (g/L)	/	/	2.23	/	/	0.77 - 3.03
Immunoglobulin M (g/L)	/	/	0.53	/	/	0.58 - 2.41
Complement C3 (g/L)	/	/	0.61	/	/	0.89 - 1.87
Complement C4 (g/L)	/	/	0.12	/	/	0.17 - 0.38
ED - emergency department. ICU - Intensive care unit. / - not determined.						

Combination of haemolytic anaemia, thrombocytopenia and oliguric renal failure was highly suspicious for thrombotic thrombocytopenic purpura (TTP) or haemolytic-uremic syndrome (HUS) as the most prominent forms of thrombotic microangiopathy (TMA). Because of that, simultaneously with additional diagnostic procedures, urgent therapeutic plasma exchange (TPE) was commenced. Additional laboratory determination of complement concentrations (represented in Table 1), immunology tests, tumour markers, and microbiological tests were done in our hospital, while antiplatelet antibodies and a disintegrin and metalloproteinase with a thrombospondin type 1 motif, member 13 (ADAMTS 13) activity was done in the referral laboratory. Since patient was anuric with a persistent rise in serum creatinine values (peak value of 636 µmol/L) from day 2 haemodialysis (HD) was started. In the meanwhile, some results were available: normal activity of ADAMTS 13 made the diagnosis of a TTP unlikely, all microbiological tests were negative but low complement concentrations along with acute renal failure were suggestible for atypical HUS. On day 7 renal biopsy was performed and while awaiting pathohistological diagnosis TPE and HD were continued. Renal biopsy revealed acute interstitial nephritis as a hypersensitivity reaction. Patient record was thoroughly re-checked and after detailed interview with patient, frequent use of ibuprofen in last few months was discovered. The patient was started on pulse dose of intravenous glucocorticoids for 3 days followed by peroral glucocorticoids with taper off regimen over 6 weeks. In all, 6 HD and 7 TPE were performed. With glucocorticoids patient condition improved and on discharge serum creatinine was 94 µmol/L without the signs of haemolysis and with normal platelet count. Table 1 provides an overview of laboratory results from admission to Emergency Department until patient discharge.

## Discussion

This case report describes a management of patient with dark brown serum/plasma coloration. Previous publications reporting such kind of colour point to methaemoglobinemia or *in vivo* hemolysis (*8*, *9*). Methaemoglobinemia is caused by change of haemoglobin iron from normal, ferrous (Fe^2+^) into ferric (Fe^3+^) form. Toxic agents like nitrates, chlorates or drugs like quinones and sulfonamides or local anesthetics cause oxidation from ferrous into ferric state (*10*).

Immunoturbidimetric analysis showed low haptoglobin concentration and confirmed *in vivo* haemolysis. Interestingly, another case of black discoloration of serum by Srivastava *et al.* described the case of a patient who was on anti-tubercular treatment, although, in their case *in vivo* haemolysis was not confirmed (*11*).

Other than methaemoglobin, dark serum coloration can be caused by presence of myoglobin or methaemalbumin, which is composed of albumin bound to oxidized free heme due to intravascular haemolysis. We could consider as limitation of the report the fact that myoglobin and/or methaemalbumin measurements were not performed in our laboratory.

## Lessons to learn from this case

In case of dark brown serum/plasma coloration, it would be advisable to:

perform biochemistry analysis of native and diluted serum,
check routine biochemistry results, specifically LD and indirect bilirubin,
communicate these findings to the clinician,
exclude suspicion to methaemoglobinemia or intravascular haemolysis by measurement of methaemoglobin and haptoglobin,
add remark on particular sample coloration on the patient report.
